# Aspirin Exacerbated Respiratory Disease: Epidemiology, Pathophysiology, and Management

**DOI:** 10.3390/medsci7030045

**Published:** 2019-03-17

**Authors:** Kevin L. Li, Andrew Y. Lee, Waleed M. Abuzeid

**Affiliations:** Department of Otorhinolaryngology: Head and Neck Surgery, Montefiore Medical Center, Albert Einstein College of Medicine, Bronx, NY 10467, USA; keli3@mail.einstein.yu.edu (K.L.L.); andlee@montefiore.org (A.Y.L.)

**Keywords:** aspirin exacerbated respiratory disease, AERD, Samter’s Triad, chronic rhinosinusitis, endoscopic sinus surgery, aspirin desensitization, nasal polyposis

## Abstract

The correlation between aspirin sensitivity, asthma, and nasal polyposis was recognized in the early 20th century. Today, this classic triad of symptoms, eponymously named Samter’s Triad, is known as aspirin exacerbated respiratory disease (AERD). Aspirin exacerbated respiratory disease affects approximately 0.3–0.9% of the general population in the USA and approximately 7% of asthmatic patients. The management of AERD is challenging as no single modality has proven to have high rates of symptom control. Consequently, disease management typically involves a multimodality approach across both medical and surgical disciplines. This review describes the epidemiology of AERD and the current state-of-the-art as it relates to the underlying pathophysiologic mechanisms of this disease process. A significant proportion of the review is focused on the appropriate diagnostic workup for AERD patients including the utility of aspirin provocation testing. The spectrum of medical treatments, including aspirin desensitization and recently introduced immunotherapies, are discussed in detail. Furthermore, surgical approaches to disease control, including advanced endoscopic techniques, are reviewed and treatment outcomes presented.

## 1. Introduction

Hypersensitivity reactions to aspirin were described as early as 1902 but it was not until 1922 that Widal et al. first described the correlation between aspirin sensitivity, asthma, and nasal polyposis [1]. Subsequently, in 1968, Samter and Beer described the full clinical characteristics of aspirin sensitivity and elucidated the classic triad of symptoms, eponymously named Samter’s Triad [1,2]. Samter’s Triad is defined as chronic rhinosinusitis with nasal polyposis (CRSwNP), bronchial asthma, and reactions to aspirin or cyclooxygenase-1 (COX-1) inhibitors [3,4,5,6]. Since its first description by Widal, there has been considerable literature published on the epidemiology, pathophysiology, and treatment of what is now termed aspirin exacerbated respiratory disease (AERD). 

## 2. Epidemiology 

A defining characteristic of AERD is an upper and lower respiratory tract reaction triggered by the ingestion of aspirin (acetylsalicylicacid, ASA) or other COX-1 inhibitors including many non-steroidal anti-inflammatory drugs (NSAIDs) [3,6]. The aspirin or NSAID-induced hypersensitivity reaction results in the rapid onset of symptoms including rhinorrhea, sneezing, nasal congestion, ocular tearing, bronchospasm, skin flushing, hives, and hypotension [7]. It is less common for concurrent respiratory and cutaneous symptoms to occur in patients [8].

It has been difficult to ascertain the prevalence of AERD in the general population [9]. Current estimates suggest that AERD affects approximately 0.3–0.9% of the general population in the USA, with a higher prevalence noted among asthmatic patients (3–20%). A 2014 meta-analysis of clinical trial data demonstrated that AERD was evident in approximately 7% of asthmatic patients [10]. The prevalence of AERD is likely higher in asthmatics who also harbor nasal polyposis with estimates ranging from 30 to 40% [11]. Interestingly, there appears to be a female predominance with incidence ratios of up to 3:2 between females and males, respectively [12]. Furthermore, females tend to have earlier symptom presentation and greater disease severity [5,6]. Generally, AERD manifests in the third or fourth decade of life [13] with much lower rates diagnosed in children [14]. There is no convincing evidence of familial inheritance in AERD [13].

## 3. Pathophysiology

**Figure 1:**
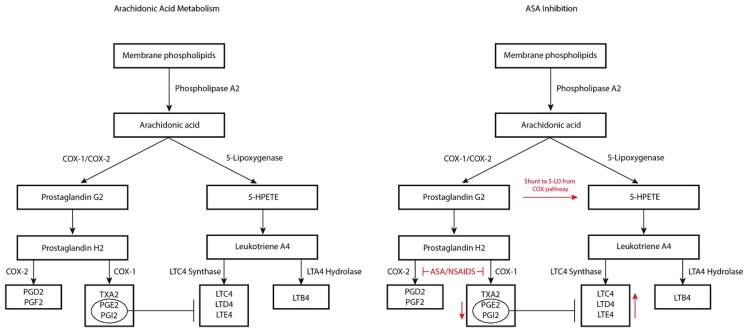
Arachidonic acid pathway (**left panel**) with associated impact of aspirin/non-steroidal anti-inflammatory disease (NSAID) therapy and inhibition (**right panel**). COX-1/2—cyclooxygenase 1/2; PGD2—prostaglandin D2; PGE2—prostaglandin E2; PGF2—prostaglandin F2; PGG2—prostaglandin G2; PGH2—prostaglandin H2; PGI2—prostaglandin I2; TXA2—thromboxane A2; 5-HPETE—5-hydroxyeicosatetranoic acid; LTA4—leukotriene A4; LTB4—leukotriene B4; LTC4—leukotriene C4; LTD4—leukotriene D4; LTE4—leukotriene E4; ASA—acetylsalicylic acid.

**Figure 2:**
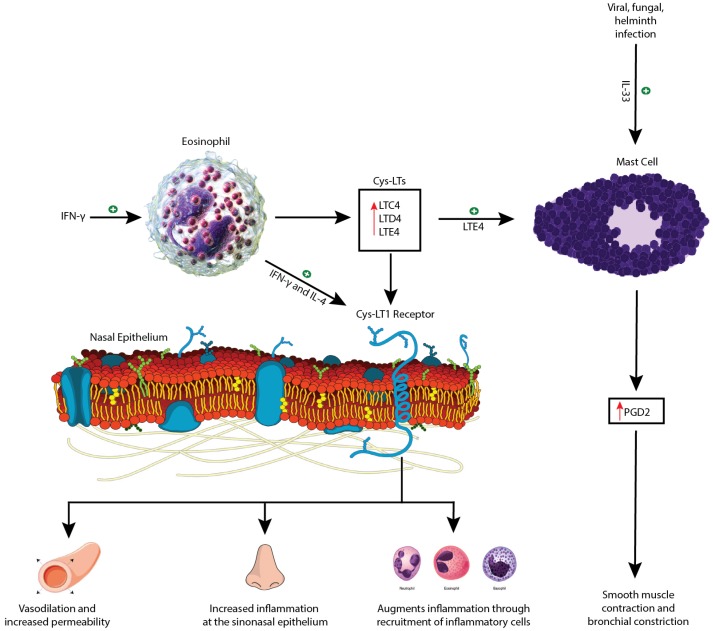
Schematic diagram depicting the role of Cys-LT1 activation and mast cell activation in pathogenesis of aspirin exacerbated respiratory disease (AERD) symptoms. INF-γ—interferon-gamma; IL-4—interleukin-4; Cys-LT—cysteinyl-leukotriene; Cys-LT1—cysteinyl-leukotriene 1; LTC4—leukotriene C4; LTD4—leukotriene D4; LTE4—leukotriene E4; IL-33—interleukin-33; PGD2—prostaglandin D2. This figure incorporates free publicly available images [41,42,43,44].

Aspirin exacerbated respiratory disease is characterized by a non-immunoglobulin E hypersensitivity reaction to ASA/COX-1 inhibitors that is commonly comorbid with but not due to underlying allergic disease [15]. Aspirin exacerbated respiratory disease is thought to be due to abnormalities in arachidonic acid biosynthesis [3,4]. Arachidonic acid can be metabolized through two different pathways: the 5-lipoxygenase (5-LO) pathway and the COX-1 pathway (Figure 1). The 5-LO pathway produces cysteinyl-leukotrienes (Cys-LTs) from arachidonic acid, specifically leukotriene C4, D4, and E4 (LTC4, LTD4, and LTE4) while the COX-1 pathway produces prostacyclins, prostaglandins, and thromboxanes. The underlying defect in AERD is thought to relate to constitutive overproduction of Cys-LTs with a concomitant decrease in downstream products of the COX-1 pathway, the latter of which have an inherent inhibitory effect on Cys-LTs [4,16,17]. The release of this physiologic brake, coupled with Cys-LT overproduction, creates a proinflammatory milieu. Indeed, Cys-LTs have been implicated in the development of rhinitis and AERD through three mechanisms (Figure 2): (1) increased vasodilation and permeability of the nasal vasculature leading to mucosal edema, manifesting clinically as nasal congestion, (2) increased inflammation at the level of the sinonasal epithelium resulting in more mucus production and rhinorrhea, and (3) augmented inflammation through the recruitment of inflammatory cells [16]. The elevated level of Cys-LTs found in the urine, sputum, exhaled breath and peripheral blood of AERD patients supports this theory [3,16]. However, there is still ongoing investigation into the mechanism underlying the constitutive overproduction of Cys-LTs.

To this end, Steinke et al. have further elucidated the roles of interferon-gamma (IFN-γ) and interleukin (IL)-4 in the pathogenesis of AERD. The cytokine milieu in AERD is notable for elevated levels of IFN-γ as compared to asthmatic or eosinophilic sinusitis. This is evidenced by the increased levels of IFN-γ mRNA transcripts and protein. Interferon-gamma is typically associated with a lymphocyte T helper 1 (Th1) response, and the authors postulate that these Th1 cells act to prevent the IgE class-switch recombination, possibly explaining the lack of allergy and atopy in AERD patients [18]. The increased IFN-γ has also been shown to stimulate differentiation of eosinophils through interferon consensus sequence binding protein, a transcription factor, leading to a dramatic upregulation in the number of infiltrating eosinophils [19]. These IFN-γ differentiated eosinophils also have significantly increased levels of LTC4 synthase (LTC4S), possibly explaining the increased levels of Cys-LTs in AERD. Moreover, eosinophils also secrete numerous cytokines and chemokines including IL-4. Both IL-4 and IFN-γ have also been shown to upregulate the Cys-LT1 receptor on multiple cell lines including eosinophils and mast cells (Figure 2) [18,20]. Therefore, IL-4 and IFN-γ both have a role in the constitutive overproduction of Cys-LTs and the overexpression of the Cys-LT1 receptor observed in AERD.

There is growing evidence that AERD may also involve an innate Th2 mucosal immune response and that this response is distinct from allergen-specific etiologies evidenced by AERD occurrence in non-atopic patients who paradoxically show elevated levels of total serum IgE [5,21]. Though there is an eosinophilic predominance in AERD, mast cells may be playing a central role in the observed hypersensitivity reactions. The role of mast cells was first suspected during oral aspirin challenges where a subset of AERD patients showed substantial reductions in FEV1 despite prophylaxis with a Cys-LT1 receptor antagonist while simultaneously showing increased levels of tryptase, a marker of mast cell activation. It has been found that the level of tryptase is inversely correlated with the change in FEV1, and activated mast cells release a host of inflammatory mediators such as prostaglandin D2 (PGD2), which induces inflammation of the respiratory epithelium through recruitment of eosinophils and Th2 cells and also harbors highly bronchoconstrictive properties. Moreover, since these patients were given Cys-LT1 receptor antagonists, the results point towards a potential function for Cys-LTs at other receptors [22,23,24].

There is increasing evidence for the role of alarmin cytokines such as IL-25, thymic stromal lymphopoeitin (TSLP), and IL-33 in the pathogenesis of the Th2 immune response through activation of group 2 innate lymphoid cells (ILC2s) [25]. Eastman et al. previously demonstrated that ILC2s are both recruited to the nasal mucosa by COX-1 inhibitor induced reactions in AERD patients and are directly correlated with symptom severity [26]. Bucheit et al. found that TSLP also activates mast cells and generates PGD2 in vivo, and in combination with IL-33, led to a synergistic increase in PGD2 production [27]. Interleukin-33 is known to induce activation of mast cells and is typically released from necrotic cells, but infections due to viruses, fungi, and helminthes have also been shown to release IL-33 from epithelial cells [28,29,30]. Moreover, surgically removed nasal polyps in AERD patients were found to have substantially more IL-33 expression than baseline. Cys-LTs were also found to induce IL-33 expression in murine models, and Pan et al. found that IL-33 stimulates mast cells to generate PGD2, thromboxane B2 (TXB2), and Cys-LTs, and requires COX-1 activity. This suggests that IL-33 could be a bridge between the Cys-LT overexpression and mast cell activation that is typical in AERD and may be a target for future pharmacotherapies [31]. Liu et al. also found that LTE4 is responsible for activation of mast cells through an IL-33 dependent pathway. Previously, LTE4 has been found to cause accumulation of eosinophils, basophils, and Th2 lymphocytes and can directly stimulate Th2 lymphocyte cytokine production [32,33]. Leukotriene E4 stimulation of mast cells has also been shown to substantially upregulate production of PGD2 through both a Cys-LT receptor pathway and a peroxisome proliferator-activated receptor-gamma (PPAR-γ) dependent pathway leading to upregulation of COX-2 (Figure 2) [34].

Laidlaw et al. found that platelet-adherent leukocytes are also effectors of AERD and lead to increased Cys-LT levels. They noted that platelet-adherent eosinophils, neutrophils, and monocytes were markedly increased in AERD patients relative to aspirin-tolerant controls and that urinary LTE4 correlates strongly with the frequency of platelet-adherent neutrophils, eosinophils, and monocytes. Moreover, their experiments found that adherent platelets expressed more than half of the peripheral blood granulocyte LTC4S activity [35]. Previous studies have also shown that activated platelets release arachidonic acid in large quantities and augment 5-LO function through the release of granulocyte macrophage-colony-stimulating factor (GM-CSF) [36,37]. Therefore, the authors concluded that platelets are likely contributing to the basal Cys-LT levels and increased levels of LTC4S found in AERD.

Finally, lipoxins also play an important role in the pathogenesis of AERD [35]. Lipoxins are endogenous anti-inflammatory mediators that typically act to inhibit inflammomodulatory cells and downregulate expression of proinflammatory cytokines such as IFN-γ, IL-5, IL-6, etc. by competing competitively at the Cys-LT1 receptor. Two important lipoxins, LXA4 and LXB4, are generated as a product of arachidonic acid metabolism. It is interesting to note that although there are upregulated Cys-LT1 receptors in patients with AERD, there is a simultaneous downregulation in the production of lipoxins, leading to inadequate competition for receptors with the Cys-LTs [38,39]. Therefore, in AERD, there may be an underlying dysregulation causing deficiency of lipoxins, contributing to the Cys-LT-driven pathophysiology [40].

The ingestion of ASA or COX-1 inhibiting NSAIDs by AERD patients further skews Cys-LT production through the inhibition of the COX-1 pathway leading to further shunting of products down the 5-LO pathway [4] (Figure 1). This is evidenced by detection of 5-LO pathway enzyme upregulation in the lungs, sinuses, and nasal polyps in AERD patients, thought to be mostly due to the infiltrating eosinophils and resident mast cells [17]. Furthermore, PGE2 normally has inhibitory effects on eosinophils and mast cells, preventing Cys-LTs from being released. ASA inhibition of PGE2 production additionally skews arachidonic acid production towards the 5-LO pathway.

AERD symptoms tend to develop gradually, beginning with the upper and lower respiratory tract. Typically, nasal congestion and rhinorrhea are the first symptoms. These symptoms persist and progress to hyposmia, nasal polyp formation, and chronic rhinosinusitis (CRS) [12]. On average, asthma developed in patients two years after the initial respiratory tract symptoms appeared, and ASA sensitivity developed within five years of onset [5].

## 4. Diagnostic Workup

The diagnosis of AERD is made through clinical suspicion and appropriate testing. AERD is suspected if patients have historical upper or lower airway clinical symptoms after ingestion of ASA or NSAIDs, chronic nasal obstruction and watery rhinorrhea, or severe asthma attacks requiring hospitalization with no apparent trigger [45]. Additionally, clinical signs such as nasal polyposis or radiologic findings such as pansinusitis on computed tomography (CT) raise the suspicion for AERD [6]. However, definitive diagnosis is only achieved through ASA provocation testing. The goal of provocation testing is to generate a hypersensitivity reaction in a safe, controlled environment with increasing doses of ASA. These hypersensitivity reactions consist of a constellation of possible symptoms including nasal and ocular itching, sneezing, conjunctivitis, wheezing, coughing, chest tightness, and a drop in forced expiratory volume (FEV1). Additional non-classical symptoms such as laryngeal tightening, stridor, vomiting, urticaria, and angioedema are also possible [13].

There are four types of ASA provocation tests: oral, bronchial, nasal, and intravenous [46]. Oral provocation testing is most commonly used in the US and has a higher sensitivity as compared to the bronchial test. The typical dose of ASA for oral provocation is from 30 to 150 mg (average 60–75 mg) [6]. The bronchial provocation test uses an inhaled L-lysine-ASA and is safer with fewer systemic reactions and is faster to perform compared to the oral challenge. Nasal provocation, typically used in Europe, also uses l-lysine-ASA and is usually reserved for patients who mainly have nasal symptoms or severe asthma contraindicating use of oral or bronchial provocation [46]. Intravenous provocation testing is rarely used outside of Japan. As oral provocation is the most commonly used, its testing protocol will be discussed below.

Provocation testing is classically carried out though accelerated regimens performed over the course of a single day are increasingly being used. In the classic two- or three-day Scripps protocol, a baseline forced expiratory volume in one second (FEV-1) is measured on Day 1 and the challenge is carried out if FEV-1 is at least 70% of the predicted value. Current provocation challenges are commonly preceded by pretreatment with one week of leukotriene modifiers such as montelukast or zileuton. This is due to their efficacy in decreasing the occurrence of severe lower respiratory reactions without inhibiting upper respiratory symptoms. Initial dosing for AERD patients is typically 20 to 40 mg and most bronchial and naso-ocular reactions occur in the dose range of 45 to 100 mg and typically appear within 30–60 min of dosing [47]. A typical oral ASA challenge usually follows a sequential dosing regimen of 30, 45, 60, 100, 150, and 325 mg spaced apart by 3 h. The larger 650 mg dose was found to not elicit additional reactions and has been discontinued from protocols [47].

Forced expiratory volume in one second is measured every 30 min up to 120 min after final dosing and patients are observed for the hypersensitivity reactions mentioned above. A positive reaction is defined as either a decrease in FEV-1 greater than 20% of baseline or if severe extrabronchial hypersensitivity reactions such as profound rhinorrhea and nasal blockade appear, even without a drop in FEV-1 below 20% of baseline. A negative reaction is defined as reaching the maximum dose of ASA without a drop in FEV-1 greater than 20% of baseline or if hypersensitivity symptoms do not appear [46,48]. Provocation tests can also measure urinary LTE4 levels which are correlated with severity of ASA reaction [49]. Increased levels of urinary LTE4 itself is not sufficient for diagnosis of AERD, but when elevated in the context of clinical parameters such as asthma exacerbations and nasal polyposis it nearly doubles the odds of AERD diagnosis [50].

A newer modified challenge protocol utilizes nasal ketorolac before oral ASA challenge and has been shown to be faster than traditional challenge, while still being safe and effective. On Day 1, this challenge protocol gives four escalating doses of ketorolac tromethamine given as nasal sprays 30 min apart with measurement of FEV1 and peak nasal inspiratory flow (PNIF). If symptoms appear, they are treated and the provoking dose is repeated. If no reaction occurs, one hour is allowed to pass before proceeding to oral ASA challenge. The first dose of ASA given is 60 mg and the patient is subsequently monitored for 90 min, wherein if no reaction is elicited, the 60 mg dose is repeated and the patient is monitored for another 90 min. On Day 2, patients are given a 150 mg and a 325 mg dose of ASA spread apart by 3 h. However, if these patients had a reaction to the second 60 mg dose on Day 1, another 60 mg dose is given before proceeding to the 150 mg and 325 mg ASA doses. Most patients finish ASA challenge by the early afternoon on Day 2, considerably faster than traditional ASA challenge testing [51,52]. New ASA challenge protocols are continuing to be developed and increase the efficiency of diagnosis. For example, DeGregorio et al. recently demonstrated that a one-day ASA challenge utilizing a 90-min dose escalation protocol at a starting dose of 40.5 mg was effective in desensitizing AERD patients with stable asthma and baseline FEV-1 greater than or equal to 70% [53].

## 5. Medical Treatment

The treatment of AERD currently incorporates an algorithm of multiple medical and surgical modalities that progress in a stepwise manner. Treatments include the use of oral and inhaled corticosteroids, leukotriene modifiers, ASA desensitization, and endoscopic sinus surgery [15].

### 5.1. Corticosteroids

Corticosteroids have been a mainstay therapy for aspirin-tolerant asthma and, although the pathophysiology differs from AERD, both inhaled and systemic corticosteroids have been found to help with subjective and objective symptoms. Specifically, it is useful in treating the symptoms of rhinosinusitis associated with aspirin hypersensitivity. Intranasal corticosteroids like fluticasone propionate have been shown to decrease the number of inflammatory cells including eosinophils and mast cells [54]. A 1997 double-blind crossover, placebo-controlled study focused on the effect of inhaled fluticasone propionate on chronic eosinophilic rhinosinusitis in AERD patients. Outcomes were measured by metrics such as nasal inspiratory peak flow and symptom scores (0–3 points) for morning and evening nasal congestion, rhinorrhea, sneezing and loss of smell. On the last day of the treatment period, l-ASA challenge was repeated. This study found the beneficial effects of fluticasone propionate appeared during the first week and showed a statistically significant increase in nasal inspiratory peak flow and a statistically significant decrease in the nasal symptom scores. The authors also found that fluticasone propionate completely prevented ASA-precipitated nasal reactions in 8 of 13 participants as measured by negative ASA provocation tests in previously positive individuals compared to 2 of 12 in the placebo arm. These results suggest that fluticasone propionate and other topical glucocorticoids are effective in treating rhinosinusitis in AERD [54]. Regardless of route, long-term corticosteroid use is associated with many negative side effects including endocrine, electrolyte, musculoskeletal and neurological disorders [55]. Therefore, other treatment modalities are used in order to reduce the dosage of corticosteroids necessary [56].

### 5.2. Leukotriene Modifiers

Leukotriene modifiers have also been widely used to treat aspirin-sensitive asthma, and due to the dysregulation of the 5-LO pathway in AERD, these drugs have become an integral treatment option [56]. Typically, anti-leukotrienes such as montelukast work at the level of the Cys-LT1 receptor, acting as a competitive antagonist. This directly leads to decreased production of Cys-LTs, compared to corticosteroids, which do not directly affect the synthesis of leukotrienes [57].

A 2002 multicenter randomized, double-blind, placebo-controlled trial studied the efficacy of montelukast as an additional treatment to AERD in 80 patients, most of whom were already treated with moderate to high doses of corticosteroids. The authors measured FEV-1 and peak expiratory flow rate in addition to asthma symptoms and quality-of-life metrics before and after treatments. The authors noted that improvement from montelukast was observed after one day of treatment. At the end of the trial, FEV-1 showed a statistically significant improvement of 10.2% on average, and patients had an improved peak expiratory flow rate difference of 28 L/min in the morning and 23.1 L/min in the evening. The montelukast arm also showed statistically significant decreases in the number daytime asthma symptoms (12.7%), rescue inhaler use (27.7%), nocturnal awakenings, (35%) and asthma exacerbations (54%). Finally, these patients also experienced significant improvement in the pooled asthma specific quality-of-life questionnaire score. This trial successfully showed that leukotriene antagonists like montelukast improved pulmonary function and asthma control above conventional corticosteroid therapy alone and is a valuable therapy to use in combination with other drug modalities [56].

In addition to montelukast, zileuton and other 5-LO inhibitors have also been studied for therapy in AERD patients. Zileuton directly inhibits 5-LO and offers another method to decrease the production of Cys-LTs. In 1998, a double-blind, placebo-controlled crossover study evaluated the efficacy of zileuton in 40 AERD patients. The patients were well controlled on corticosteroids previously and the zileuton arm received four 600 mg doses. Outcomes measured included FEV-1, peak expiratory flow rate, beta-agonist use, and daytime and nocturnal subjective symptoms, including loss of smell, rhinorrhea, and congestion (scored 0–3). The zileuton arm showed a significant increase in the FEV-1 within hours (12.7% increase, *p* < 0.01) and this benefit lasted throughout the study period. Moreover, there was an 18 L increase in the morning peak expiratory flow rate (*p* < 0.001) compared to placebo, and beta-agonist use decreased by 0.64 puffs (*p* < 0.05). Daytime and nocturnal subjective symptoms scores did not differ significantly in this study, which the authors attribute to the well-controlled symptoms in the patient population at baseline. The authors showed that 5-LO inhibitors were an effective therapy for the treatment of AERD [58]. Interestingly, a clinical questionnaire given to AERD patients found that zileuton is very effective in reducing asthma symptoms compared to montelukast. Moreover, a subgroup analysis in patients with asthma that reported symptoms with ASA use, but were not formally diagnosed, found that zileuton led to a nearly 20% increase in FEV1, indicating that it could be used in the treatment of asthma in AERD patients [59,60]. The efficacy of zileuton over montelukast may be a consequence of its upstream inhibition of 5-lipoxygenase resulting in downregulation of all downstream Cys-LTs, whereas cys-LT1 receptor antagonists, like montelukast, would not significantly affect LTE4 [15,58]. Simultaneous use of a 5-LO inhibitor and a Cys-LT1 receptor antagonist has been suggested but not formally studied at this point [61].

Leukotriene modifiers (both montelukast and zileuton) have also been shown to provide a degree of protection during ASA challenge testing. A 2006 study reviewed the records of 676 patients who completed oral ASA challenges and found that patients taking leukotriene modifiers had significantly less (10–20%) decline in FEV-1 post-provocation. The authors also found that pre-treatment with leukotriene modifiers resulted in less severe asthmatic reactions and a decrease in lower respiratory tract symptoms, possibly due to the abundance of Cys-LT1 receptors in the lower airways compared to the upper airways [61,62]. Consequently, pre-treatment with leukotriene modifiers has been integrated into many ASA challenge protocols.

### 5.3. Aspirin Desensitization

Corticosteroids and leukotriene modifiers are the first line therapies used to treat AERD. However, if these are insufficient in controlling symptoms, ASA desensitization can provide added benefits. Some authorities believe that all AERD treatment plans should utilize desensitization [13,63]. The exact mechanism by which ASA desensitization helps control symptoms is currently unknown, but there has been evidence that it decreases IL-4 and STAT6 transcription, decreases production of PGD2, LTE4, and IFN-γ, and decreases the density of Cys-LT receptors [13,17,64,65,66].

There are multiple protocols developed for ASA desensitization, but typically, ASA desensitization occurs by bringing a patient to a well-equipped clinic and slowly administering increasing doses of ASA until a reaction is elicited [64]. Then, a maintenance dose of 650 mg twice a day is established for continual treatment. If tolerated well, after 6 months, it is reduced to 325 mg twice a day [13,47]. ASA desensitization, followed by either 325 mg twice a day or 650 mg twice a day post-endoscopic sinus surgery with polyp removal is now the standard of care for AERD patients. Typically, the ASA desensitization and treatment is started three to four weeks after the first sinus surgery [15,67].

### 5.4. Monoclonal Antibodies

Monoclonal antibodies are becoming increasingly popular as a potential therapy in the treatment of AERD. Omalizumab is a recombinant antibody originally designed for treatment of asthma through binding of IgE receptors on mast cells and basophils [68]. Omalizumab has been shown to have mixed efficacy in studies; some authors have found that it displayed rapid clinical effectiveness in reducing mast cell activation and leukotriene overproduction, while others have found that the reduction is not statistically significant [68,69].

In 2013, Gevaert et al. published a randomized, double blind, placebo-controlled trial studying omalizumab in 24 patients with asthma and CRSwNP. The authors had primary end points of polyp size reduction as measured by a total nasal endoscopic polyp score (TPS, scored 0–4). Secondary endpoints were improvement in clinical symptoms measured by Lund-MacKay scores and quality-of-life questionnaire scores including the Short Form Health Questionnaire (SF-36), Rhinosinusitis Outcome Measuring Instrument (RSOM-31), and the Asthma Quality-of-Life Questionnaire (AQLQ). In the omalizumab arm, polyp size and TPS score was significantly reduced by the end of the trial (−2.67, *p* = 0.001), and Lund-Mackay scores were significantly improved as well (17.6 to 13.6, *p* = 0.02) compared to placebo. The omalizumab arm also had significantly improved SF-36 scores for physical health (*p* = 0.02) but not mental health. Rhinosinusitis Outcome Measuring Instrument scores showed significant improvement in sleep (*p* = 0.03) and general symptoms (*p* = 0.01). The mean AQLQ score increased 0.81 (*p* = 0.003). This study demonstrates that omalizumab is capable of improving both disease severity and quality-of-life metrics. Unfortunately, this study primarily focused on CRSwNP and not on AERD. However, within their study group, 12 of 24 patients were given a diagnosis of aspirin hypersensitivity based on medical history. These patients were not challenged with ASA so a definitive diagnosis of AERD could not be given. While further investigation is needed, this could signify a role for omalizumab in AERD patients [70].

In a 2016 prospective cohort study, Hiyashi et al. found that omalizumab administration produced a significant decrease in concentration of urinary LTE4 and a PGD2 metabolite, 9α,11β-prostaglandin F2 (PGD2M) in a post-surgical AERD population and helped to ameliorate upper and lower respiratory tract symptoms, possibly due to mast cell stabilization. Twenty-one patients were studied and, following administration of omalizumab, there was a 76.2% decrease in urinary LTE4 (*p* < 0.001) and an 89% decrease in PGD2M (*p* = 0.002). In addition, they also found a 36.3% drop in eosinophil count (*p* = 0.002) and a significant decrease in the number of exacerbations (*p* = 0.002) and hospitalizations (*p* = 0.001) in a 12-month period. Finally, the Visual Analog Scale (VAS) score was significantly improved for nasal congestion, anterior rhinorrhea, anosmia, dyspnea, wheezing, and cough (*p* < 0.001). The authors demonstrated that omalizumab improved both upper and lower respiratory tract symptoms, which was correlated with the decrease in urinary LTE4 levels [69]. However, this trial was neither randomized nor placebo-controlled, so future studies are needed to verify the potential value of omalizumab in the treatment of AERD.

Mepolizumab is another monoclonal antibody that has been proposed as therapy for AERD. Mepolizumab targets IL-5 and was originally designed to treat eosinophilic asthma, but Gevaert et al. found in a randomized, double-blind placebo-controlled study in CRSwNP patients that injection of two 750 mg doses significantly reduced the total polyp score (−1.30, *p* = 0.028) and showed improved CT scan results in 12 of 20 patients when reviewed by three separate raters (Fleiss κ = 0.679) [71,72,73]. Again, this study was not specifically designed for AERD patients but 5/20 patients in the treatment group had aspirin sensitivity. Bachert et al. found in a similar randomized, double-blind, placebo-controlled study with 107 CRSwNP patients that 750 mg of mepolizumab every four weeks for six doses resulted in a significant reduction in the endoscopic nasal polyposis score (50% of patients improved by >1 point), and the odds ratio of having a reduction in total endoscopic nasal polyp score was high (6.6, *p* = 0.025). The nasal polyposis severity VAS score was also significantly improved for rhinorrhea (*p* < 0.001), mucus in throat (*p* < 0.001), nasal blockage (*p* = 0.002), and loss of smell (*p* < 0.001). There was also significant improvement in the Sino-Nasal Outcome Test (SNOT-22) scores (42 to 27.1, *p* = 0.005). The authors concluded that administration of mepolizumab decreased the overall need for surgical intervention [74]. This study was also not stratified to include AERD patients and is not directly applicable to this patient population. However, a 2018 retrospective study on mepolizumab for AERD patients has shown some positive results. Fourteen AERD patients at Brigham and Women’s Hospital, Boston, MA, USA, who received at least three doses of mepolizumab were included in the study and outcomes such as absolute eosinophil count (AEC), SNOT-22, asthma control test (ACT) scores, and FEV1 were investigated. At baseline, many of these patients had already received numerous AERD treatments such as polypectomies, high-dose aspirin, and oral glucocorticoids. After receiving at least three doses of mepolizumab, the AEC decreased significantly (*p* < 0.01), SNOT-22 decreased by 17.7 points (*p* < 0.01), ACT score increased significantly by 5.1 (*p* = 0.002), but FEV1 percent predicted increased non-significantly by 6.3% (*p* = 0.16). Additionally, no patient required sinus surgery during this time and no patient needed to start or increased their dose of glucocorticoids. On the contrary, five of the seven patients on oral glucocorticoids actually reduced their doses and two of five patients on daily-inhaled corticosteroids/long-acting beta-agonists were able to reduce their doses as well. This was the first study to show the efficacy of mepolizumab in AERD patients, but future double-blinded, controlled studies are needed to confirm this data [75]. Reslizumab and benralizumab are similar agents that also target IL-5 and may have similar efficacy but further investigation is needed to fully elucidate the effectiveness for AERD patients [71].

Dupilumab is the latest monoclonal antibody studied as a therapy for CRSwNP. It is a fully humanized monoclonal antibody that acts directly against the IL-4 receptor α subunit. This in turn inhibits the action of both IL-4 and IL-13, two cytokines that are integral to the Th2-cell mediated inflammatory response. Dupilumab has already been shown to be effective in treating patients with atopic dermatitis and asthma [76]. In a multicenter, randomized, double-blind placebo-controlled parallel-group study conducted in the US and Europe, Wenzel et al. studied dupilumab treatment of CRSwNP refractory to intranasal corticosteroids alone in 104 patients, 15 of which were aspirin sensitive. They found that dupilumab resulted in significant improvements in endoscopic, radiographic, clinical, and pharmacological measures 16 weeks post-treatment. The primary end point was number of asthma exacerbations, and dupilumab showed a significantly reduced number of exacerbations compared to placebo (odds ratio 0.08, *p* < 0.001). Additionally, FEV1 improved by 0.27 L compared to placebo (*p* < 0.001) and morning peak expiratory flow improved by 34.6 L/min compared to placebo (*p* = 0.005). They also found significant improvement in quality-of-life metrics like SNOT-22 (−8.49, *p* = 0.003) with improved sense of smell, fewer symptoms of nasal obstruction and decreased nighttime awakenings. The authors demonstrated that dupilumab treatment in persistent asthma was associated with fewer exacerbations and increased objective and subjective outcome metrics. This improvement was seen in a population that was already treated with medium to high doses of inhaled glucocorticoids and long-acting beta agonists, further suggesting that blocking IL-4 and IL-13 signaling results in an improvement in nasal polyposis, asthma, and improved upper and lower respiratory tract inflammation [77]. A substudy on AERD patients in a phase II trial of dupilumab by Mullol et al. showed that treatment with dupilumab produced an improvement in almost 10 items in the University of Pennsylvania Smell Identification Test (UPSIT) and led to a 30 point reduction in SNOT-22 score, as well as a 2.5 point reduction in Total Polyp Score [78]. Although there is a lack of studies specifically on AERD patients, future studies may prove dupilumab to be a valuable therapeutic agent [76].

## 6. Surgical Procedures and Outcomes

The role of endoscopic sinus surgery (ESS) can play an integral part in treatment of AERD patients, having a role in decreasing disease burden itself while providing an opportunity for more effective medical treatment [79]. Surgical approaches are targeted towards optimizing the ventilation and drainage of the paranasal sinuses through the widening of the sinus ostia and removal of inflamed bone and soft tissue components. Critically, surgery also enhances the delivery of topical corticosteroids into the paranasal sinuses thereby improving control of inflammation at the level of the sinus epithelium. Computational fluid dynamic models have shown generally enhanced delivery of sinus rinses into the paranasal sinuses after ESS with one study predicting a 10-fold increase in the number of nebulized particles deposited within the maxillary sinus after uncinectomy and antrostomy [80,81]. Furthermore, a separate study evaluating a cohort of 28 patients with confirmed AERD by aspirin challenge, found that that AERD patients were less reactive to an aspirin challenge 3–4 weeks after endoscopic sinus surgery with 43% (*p* < 0.001) having no detectable reaction [82].

AERD patients are known to be a particularly difficult patient population to successfully manage and in whom single modality treatment is rarely successful with quoted failure rates of up to 90% for standard endoscopic sinus surgery [3,83]. Most commonly, dual therapy with a surgical approach combined with ASA desensitization is implemented rather than single modality therapy. The use of sinus surgery leads to decreases in symptomatic severity providing an optimal window in which to proceed with additional treatments including aspirin desensitization and therapy. The ideal time period post-surgical intervention has been postulated as 2–4 weeks [84].

The combined method has shown an improvement in both subjective and objective measures of sinonasal outcomes as measured by SNOT scores [63,85]. In the retrospective review conducted by Cho et al. examining outcomes of aspirin desensitization post-ESS in AERD patients, the authors noted that SNOT-22 scores significantly decreased immediately postoperatively at one week (*p* = 0.042) and four weeks (*p* = 0.046) and continued to remain low through the 30-month post-desensitization follow up period. Endoscopic polyp grade also decreased significantly in the post-operative period (*p* < 0.001) and remained low for up to 30 months post-desensitization with no significant recurrence of polyp burden [85]. This may imply that long-term aspirin desensitization may prevent or slow the progression of the inflammatory process within the sinuses [86]. In a study examining long-term clinical outcomes of ASA desensitization therapy, 92 patients completed a questionnaire regarding nasal symptoms during/after ASA desensitization therapy that was initiated 5–10 years prior with 68% of patients not requiring further sinus surgery and 85% of patients finding it helpful in improving airway disease and quality of life. Interestingly, within this same cohort, ASA therapy did not reduce the total number of sinus surgeries (*p* = 0.56) or delay time to the next sinus/polyp surgery (*p* = 0.27) in those that required further interventions [87]. However, another study found that after surgery, ASA desensitization and long-term ASA therapy reduced reoperative intervention from an average of once every three years to once every 10 years. This further emphasizes the importance of ASA desensitization in combination with surgery [88].

Compared to patients with non-AERD CRSwNP, AERD patients tend to have more severe sinonasal symptoms, as measured on validated symptom score surveys, and a higher incidence of recurrent polyposis up to as high as 90% [83], resulting in higher rates of surgical intervention [45,63,89]. On average, AERD patients undergo 2.6 endoscopic sinus surgeries during their lifetime and tend to be younger at the time of first surgery [90]. When comparing AERD CRSwNP to non-AERD CRSwNP (asthma + CRSwNP and CRSwNP alone), one study—the design of which was predicated on the interpretation of disease severity based on a diagnostic CT scan—demonstrated that 66% of AERD CRSwNP patients were classified as having severe sinus disease compared to 23% and 10% in the other groups, respectively (*p* < 0.001) [90]. In addition to more aggressive symptoms, patients with AERD have significantly worse surgical outcomes compared to patients with non-AERD sinus disease [91]. In a cohort of 549 patients with nasal polyposis undergoing ESS, patients with AERD had increased odds of requiring a second surgery for recurrence compared to patients without asthma or asthma alone (odds ratio 2.7, *p* < 0.01) [83]. There has been no conclusive randomized trial data driving the choice for surgical treatment of AERD, and as such, multiple surgical techniques and procedures have been developed to treat CRS and AERD refractory to medical treatment [63,92]. In the past few decades, the surgical approach has evolved from invasive procedures to minimally invasive endoscopic mucosal-sparing surgeries [93]. Some authors suggest a graduated approach to surgical intervention that is tailored to the patient’s disease process and severity. Factors that help with this personalized approach include the patient’s disease history, nasal endoscopy, and CT findings [92,94].

### 6.1. Functional Endoscopic Sinus Surgery

The standard of management for CRS and AERD refractory to medical treatment is functional endoscopic sinus surgery (FESS) [95,96]. The primary goal is to clear diseased tissue within the sinonasal cavities under endoscopic guidance, to re-establish ventilation and drainage via normal physiologic routes, and to optimize the delivery of topical therapeutics, particularly corticosteroids, to the epithelium of the paranasal sinuses [97]. For patients with refractory CRS after initial primary FESS, there are advanced surgical procedures used to treat the frontal, maxillary, or ethmoid sinuses which are discussed below [93].

Functional endoscopic sinus surgery alone in the noncomplicated CRS patient has yielded significant improvements in quality-of-life metrics but, in AERD patients, the role of surgery is less definitive. Surgical intervention alone in the AERD cohort has shown initial improvements in symptoms and disease control but with high rates of recurrence and need for subsequent surgeries [83]. In one study looking at complete ESS, entailing surgical access to all paranasal sinuses, versus targeted ESS, which involves treating only those sinuses that appear diseased on preoperative CT imaging, ASA sensitivity was an independent predictor for complete ESS. Furthermore, complete ESS showed greater improvements in quality-of-life metrics compared to the targeted therapy cohort [98].

The true impact of ESS within the AERD cohort is best characterized when used in conjunction with aspirin desensitization as this is the optimal use scenario based on our current understanding of disease management. In a retrospective review of 32 patients undergoing complete ESS followed by aspirin desensitization therapy, only three patients (9.4%) needed revision sinus surgery within the 30 month follow-up period—one of these patients had stopped ASA therapy during the course of the study [89]. Furthermore, overall SNOT-22 scores showed significant improvement one month postoperatively compared to preoperative baseline (47.0 vs. 15.2, *p* < 0.001) and remained statistically unchanged during the 30 month follow-up period after ASA desensitization was initiated, consistent with previous studies [85,89]. Complete ESS has a role in treatment of AERD patients with significant initial improvement in disease burden and quality-of-life measures, but the evidence thus far suggests that combining complete ESS with ASA desensitization post-operatively produces the greatest effect on disease control.

### 6.2. Endoscopic Modified Lothrop Procedure/Draf 3

Frontal sinus surgery has high treatment failure rates and often requires revision surgery. One study found that ethmoidectomy without frontal sinusotomy could be used as a first-step procedure for treatment of chronic frontal sinusitis in patients who are already on maximal medical therapy. However, nasal polyposis and ASA sensitivity were independent risk factors predicting failure in those who underwent treatment with this more conservative ethmoidectomy alone approach [99]. This suggests that ethmoidectomy alone may be inadequate for treatment of frontal sinus disease within high-risk recurrent groups such as AERD patients.

An alternative to salvage failed FESS is the endoscopic modified Lothrop procedure (EMLP), also known as the Draf 3 procedure [93,95]. Endoscopic modified Lothrop procedure results in a large common drainage pathway for both frontal sinuses by removing the medial frontal sinus floor bilaterally to the orbits laterally and resecting the superior nasal septum and intersinus septum [94,100]. Another advantage is the ability for sinus rinses to penetrate and distribute within the frontal sinus through the new common pathway with mathematical models showing a significantly increased penetration of sinus rinses into the frontal sinuses after EMLP [101]. Naidoo et al. found in a retrospective cohort study that EMLP allows for increased delivery of topical steroids to control local mucosal inflammation as well as increasing the ventilation into the frontal sinuses. Unfortunately, there appears to be a subset of patients that have exacerbations despite long-term medical therapy [96].

A 2018 meta-analysis by Abuzeid et al. showed that EMLP improved symptoms in 82.3% of patients with 75.9% of patients reporting improvement when EMLP was used as a salvage surgery after failure of primary FESS. Interestingly, the authors found that patients with ASA sensitivity and asthma appeared to have a lower incidence of reoperation, which was attributed to possible evolution to a more aggressive surgical technique based on an understanding that AERD patients were at higher risk of surgical failure [95]. Nevertheless, failure rates in EMLP have been cited as 5–21% across diverse patient pathologies, with many of these cases then requiring a revision EMLP or frontal sinus obliteration [91,96,102]. Failure typically occurred secondary to recurrent polyposis or stenosis of the ostium [91]. Generally, EMLP is considered a safe and efficacious surgery in the modern era. Furthermore, EMLP provides an attractive option for revision surgery as it does not preclude additional surgical options should patients develop refractory disease [95,102].

### 6.3. Complete Total Ethmoidectomy with Mucosal Stripping

In AERD patients, complete total ethmoidectomy with mucosal stripping or nasalization has been shown to have greater efficacy than conventional ethmoidectomy [93]. Nasalization involves the systematic removal of all the bony lamellae and mucosa in the ethmoid sinuses followed by maxillary antrostomy, sphenoidotomy, frontal sinusotomy, and middle turbinectomy [93].

Eloy et al. found that patients who underwent nasalization showed superior improvement in nasal symptoms compared to those who underwent a standard ESS and the outcomes were more durable. Specifically, olfactory improvement in the nasalization arm lasted for three years compared to only two years of symptom improvement in the ethmoidectomy group [93]. Jankowski et al. has also shown that nasalization is superior with regards to overall symptoms, disease severity as measured on CT, and endoscopic appearance of the post-operative mucosa. Immediately post-operative, patients were started on nasal lavages and local beclomethasone sprays. Critically, nasal polyp recurrence rate was 22.7% in the nasalization arm versus 58.3% in patients undergoing traditional ethmoidectomy. When performed by a skilled sinus surgeon, nasalization was not found to be more hazardous than standard ethmoidectomy [103].

## 7. Conclusions

Treatment of CRSwNP in the setting of AERD poses a challenging problem within the otolaryngology community. With higher rates of refractory disease despite optimal medical and surgical treatment options, finding the right combination of treatment modalities to help improve symptom control and quality-of-life within this patient population continues to be an active area of research interest. Further improvements in disease control will likely hinge on modification of the underlying inflammatory milieu at the level of the sinonasal epithelium. This will involve the continued development and introduction of biologic immunomodulators for clinical use. Advanced FESS procedures will also play an increasing role in optimizing the delivery of medical therapies and directly modifying the levels of inflammation in the sinuses. Continued advances in these areas, and a better understanding of the ideal timing for specific interventions, will lead to an era of patient-specific treatment and, potentially, improved long-term disease control.
